# Characterization of Spectrin Family Genes and Their Evolutionary Roles in Domestication and Breeding of the Silkworm *Bombyx mori*

**DOI:** 10.3390/insects16060556

**Published:** 2025-05-24

**Authors:** Kunpeng Lu, Chengyu Zhan, Jianghong Shen, Chao Zhi, Jun Deng, Kerui Lai, Minjin Han, Hai Hu, Xiaoling Tong, Fangyin Dai

**Affiliations:** State Key Laboratory of Resource Insects, Key Laboratory of Sericultural Biology and Genetic Breeding, Ministry of Agriculture and Rural Affairs, College of Sericulture, Textile and Biomass Sciences, Southwest University, Chongqing 400715, China; lukunpeng@swu.edu.cn (K.L.); zhancyres@email.swu.edu.cn (C.Z.); sjh0522@email.swu.edu.cn (J.S.); chaozhi@email.swu.edu.cn (C.Z.); dhl1_1@outlook.com (J.D.); laijirong@outlook.com (K.L.); minjinhan@126.com (M.H.); huhaiswu@163.com (H.H.); xltong@swu.edu.cn (X.T.)

**Keywords:** spectrin family genes, silkworm, gene structure optimization, domestication, breeding

## Abstract

The spectrin family genes play critical roles in cytoskeletal organization and cellular integrity, yet their evolutionary and functional significance in non-classical model organisms remains poorly explored. Here, we systematically identified and characterized spectrin family genes in the silkworm *Bombyx mori*. Genome-wide analysis identified 17 predicted spectrin genes, which were integrated into eight optimized members through transcriptome-guided structural refinement. Multi-species genomic analysis revealed 8, 23, and 24 spectrin family genes in *Drosophila melanogaster*, *Mus musculus*, and *Homo sapiens*, respectively. Phylogenetic analysis revealed conserved clades across insects and mammals, with gene family expansions in vertebrates. Spatiotemporal expression profiling demonstrated ubiquitous expression of these genes during silkworm development. Population genomic analyses detected strong selection signatures in *BmTrio* during domestication and implicated *BmBeta_spc* as a candidate gene for silk yield enhancement in Chinese-improved strains (CHN-I). Expression profiles of parental strains and F_1_ offspring from a commercial hybrid cross (Jingsong × Haoyue) revealed *BmBeta_spc* expression correlating with heterosis in silk yield traits. This study elucidates the characterization and functional relevance of silkworm spectrin genes, providing insights into their roles in domestication and breeding.

## Simple Summary

Spectrin family genes are crucial for maintaining cell structure, but their roles have mainly been studied in neural and muscle systems of common model organisms. Here, we identified 8, 23, 24, and 8 spectrin family genes in the genomes of *Drosophila melanogaster*, *Mus musculus*, *Homo sapiens*, and the silkworm *Bombyx mori*, a newly established Lepidopteran model organism. Phylogenetic analysis showed that these genes are conserved across insects but expended in mammals. Transcriptome data highlighted their broad roles in silkworm growth and development. One gene, *BmTrio*, showed strong selection during silkworm domestication, potentially linked to behavioral changes like reduced activity in farmed silkworms. Another gene, *BmBeta_spc*, was associated with increased silk production in Chinese-improved silkworms and enhanced productivity in hybrid offspring. These findings deepen our understanding of spectrin family genes in non-classical model species and offer new targets for improving silk yields through molecular breeding.

## 1. Introduction

Spectrin family proteins are characterized by spectrin repeats (SRs) and other conserved domains, including calponin homology (CH), EF hands, and calcium-binding motifs [1]. These proteins serve as key components of the membrane cytoskeleton, mediating actin filament bundling, crosslinking, and binding [2,3]. Initially identified in erythrocyte ghost membranes [4], spectrins were later detected in non-erythroid tissues, such as neural tissues, skeletal/cardiac muscle, and the brain [5]. Core family members include *dystrophin*, *alpha-actin*, *spectrin* (*α-* and *β-spectrin*), *nesprin*, and *Macf1* (Microtubule-Actin Crosslinking Factor 1). *Macf1*, orthologous to *Drosophila Shot* (*Kakapo*) and *vab-10* in *Caenorhabditis elegans* (*C. elegans*), is known as one of the longest proteins in animal genomes.

Spectrin genes are indispensable across diverse biological processes in both vertebrates and invertebrates. In *Homo sapiens*, mutations in spectrin family genes are linked to various diseases. For instance, *dystrophin* mutations (point mutations, small insertions/deletions, and large deletions or duplications) cause Duchenne muscular dystrophy (DMD) and an X-linked muscle wasting disease [6,7]. *Actn4* mutations underlie familial focal segmental glomerulosclerosis (FSGS), a common non-specific renal lesion [8]. Heterozygous duplication of *Macf1* reduces its expression, leading to muscular dystrophy [9]. In *Drosophila*, *shot* regulates neural function, muscle attachments, and cell adhesions [10], while α- or β-spectrin deficiency disrupts presynaptic neurotransmitter release [11]. In *C. elegans*, *vab-10A* and *vab-10B* loss reduces numbers of fibrous organelles and increases epidermal thickness, respectively [12]. Collectively, these studies elucidate spectrin functions in model organisms. Nevertheless, expanding research to non-classical model species remains critical, as gene functions may diverge across taxa, and broader sampling is essential to comprehensively map functional evolution.

The domesticated silkworm, *Bombyx mori* (*B. mori*), originated from its wild ancestor, *Bombyx mandarina* (*B. mandarina*). As an economically important insect in agriculture, *B. mori* is globally cultivated for the production of silk, a renowned luxury fiber used in various fields such as textiles and biomedicine. Research on silkworm has enhanced our knowledge of insect development, genetics, as well as silkworm domestication and breeding in the past several decades [13,14,15]. However, the characterizations and evolutionary roles of spectrin family genes in silkworms remain largely unexplored.

Spectrin family genes encoding ultra-large proteins are frequently misannotated as fragmented gene models in genome assemblies, particularly in non-classical model organisms. Accurate identification of these genes through multi-omics integration is essential to enable downstream functional genomic studies. In this study, we identified the silkworm spectrin genes through genome-wide analysis and refined their structures by utilizing transcriptome evidence. We characterized protein domains and spatiotemporal expression patterns. Integrated population genomics, comparative transcriptomics, and association studies revealed a potential role of *BmTrio* in silkworm domestication and linked *BmBeta_spc* to silk yield enhancement and heterosis. These findings provide a comprehensive molecular atlas of silkworm spectrin family genes, establishing a framework for functional studies in silkworm domestication, breeding, and insect biology.

## 2. Materials and Methods

### 2.1. Genome-Wide Identification of Spectrin Repeat Domains

Whole-genome protein sequences of *B. mori* were retrieved from Silkbase (http://silkbase.ab.a.u-tokyo.ac.jp/cgi-bin/index.cgi, accessed on 19 February 2019) [16]. The Hidden Markov Model (HMM) profile of spectrin repeats (PF00435) was downloaded from the Pfam database (http://pfam.xfam.org/, accessed on 5 December 2019) [17]. Using HMMER3.2 (hmmsearch program, e-value ≤ 0.01), we identified SR-containing proteins and manually removed redundant sequences [18]. Predicted protein domains were validated via the SMART (http://smart.embl.de/, accessed on 31 July 2020) and CDD v3.18 (https://www.ncbi.nlm.nih.gov/Structure/cdd/docs/cdd_search.html, accessed on 31 July 2020) online tools [19,20]. To minimize false positives, hits with SR domain e-values ≥ 0.01 were manually excluded. The protein sequences of *Mus musculus* (*M. musculus*) and *Homo sapiens* (*H. sapiens*) were downloaded from the Ensembl database (http://asia.ensembl.org/index.html, accessed on 25 December 2019); the protein sequences of *Drosophila melanogaster* (*D. melanogaster*) were downloaded from Flybase (http://flybase.org/, accessed on 25 December 2019).

Gene structures and chromosomal coordinates were extracted from Silkbase, with chromosomal distributions visualized using TBtools v0.66831 [21].

### 2.2. Gene Structure Optimization and Protein Domain Analysis

To refine spectrin gene structures, transcriptome data (SRA accessions SRR10035668 and SRR10035660) were obtained from the Sequence Read Archive (SRA) database. The reference genome was downloaded from Silkbase [16]. Raw transcriptome reads were filtered using the fastp (-n 15 -q 20 -u 50) program and mapped to the reference genome using the hisat2 (defaulted parameters) program [22]. Transcript assembly and structural optimization were performed with Cufflinks and Cuffcompare (default settings) [23].

Open reading frames (ORFs) and protein domains were annotated using the ORFfinder (https://www.ncbi.nlm.nih.gov/orffinder/, accessed on 16 April 2020), SMART, and CDD v3.18 online tools [19,20]. Multiple sequence alignments were conducted using MAFFT v7.455 [24], and phylogenetic trees were constructed in MEGA X using the neighbor-joining method with 1000 bootstrap replicates [25].

### 2.3. Spatiotemporal Expression Analysis of Spectrin Genes

To characterize spatiotemporal expression patterns, we obtained expression data (FPKM, fragments per kilobase of transcript per million mapped reads) from the SilkMeta database (http://silkmeta.org.cn accessed on 20 December 2023) [26]. This dataset, derived from BioProject PRJNA559726 [27], includes transcriptomic data across 13 larval/pupal tissues (hemolymph, epidermis, head, testis, ovary, malpighian tubule, trachea, midgut, fatbody, and anterior/middle/posterior silk glands) and seven adult tissues (thorax, antenna, legs, wing, head, testis, and fatbody). Expression patterns were visualized via heatmaps generated with R v4.4.2 software.

### 2.4. Silkworm Rearing, Dissection, qPCR Analysis, and Silk Yield Trait Investigation

The silkworms were reared at the National Silkworm Genetic Resources Gene Bank, Southwest University, China. Improved strains, Jingsong, Haoyue, Jingsong × Haoyue (F_1_ hybrid), Furong, Xiafang, Xianghui, and 7532, were utilized for silk gland collection. Silk glands from Furong, Xiafang, Xianghui, and 7532 were dissected on day 3 of the fifth larval instar, flash-frozen in liquid nitrogen, and stored at −80 °C for RNA extraction and RNA-seq. Each RNA-seq sample pooled silk glands from at least three individuals (two biological replicates). For Jingsong, Haoyue, and F_1_ hybrids (Jingsong × Haoyue), silk glands were collected on days 1, 3, and 5 of the fifth larval instar, preserved at −80 °C, and processed for RNA extraction and qPCR (three biological replicates, ≥3 individuals per replicate).

For qPCR, total RNA was isolated using TRIzol (Simgene, Hangzhou, Zhejiang, China). RT-qPCR was performed on a qTOWER 3G system (Analytik Jena AG, Jena, Germany) with SYBR Green Pro Taq HS (Accurate Biology, Changsha, China). Relative expression levels were calculated using the 2^−∆∆Ct^ method, normalized to the eukaryotic translation initiation factor 4A gene (*KWMTBOMO02081*). The inherited expression modes of *BmBeta_spc* underlying heterosis were determined through qPCR data comparison, as described in a previous report [28].

To evaluate heterosis in silk yield traits of the Jingsong × Haoyue hybrid, parental lines (Jingsong and Haoyue) and their F_1_ offspring were reared concurrently under standardized environmental conditions (25 °C, 12 h light/12 h dark photoperiod). At the pupal stage, silk yield traits, including cocoon weight (CW), cocoon shell weight (CSW), and cocoon shell ratio (CSR; CSR = CSW/CW × 100%), were measured in the parental strains Jingsong and Haoyue and their F_1_ hybrid (Jingsong × Haoyue). Statistical significance was evaluated using the Student’s *t*-test.

### 2.5. Transcriptome Sequencing and Analysis

Transcriptome sequencing was performed on silk gland samples from Furong, Xiafang, Xianghui, and 7532. RNA-seq library preparation, sequencing, and bioinformatic workflows followed established protocols [14]. Additionally, FPKM expression profiles of the C145, Chishu, Huangbo, 305, and Chunfeng strains were obtained from SilkMeta [26]. C145, Chishu, and Huangbo are local silkworm strains; Furong, 305, and Xiafang are Chinese-improved strains (CHN-I); Xianghui, 7532, and Chunfeng are Japanese-improved strains (JPN-I). Local silkworms are defined as traditional, non-improved varieties, while improved strains refer to those selectively bred by breeders for enhanced traits.

To address technical variability across batches in RNA-seq datasets, we implemented the ComBat algorithm (R package) for empirical Bayes adjustment of FPKM expression values. Processed *BmBeta_spc* expression data were analyzed across local, CHN-I, and JPN-I groups. Tissue expression profiles of *BmTrio* in wild and domesticated (“Lao”) silkworms were retrieved from SilkMeta for comparative analysis, focusing on day 3 of the fifth larval instar. Statistical significance was assessed using the Student’s *t*-test.

### 2.6. Analysis of Genomic Variations

Genomic variations, including single nucleotide polymorphisms (SNPs), short insertions and deletions (InDels, < 50 bp), and structural variations (SVs), were obtained from the silkworm pan-genome dataset on SilkMeta [14,26]. Population genetic statistics, *F*_ST_ (fixation index) and Tajima’s D values, were retrieved from SilkMeta. SNPs, InDels, and SVs within *BmTrio* and *BmBeta_spc* loci were analyzed for divergence between subpopulations (wild vs. local, local vs. CHN-I, and JPN-I vs. CHN-I) using the chi-square test, and the *p*-values were corrected using the Benjamini–Hochberg false discovery rate (FDR) method. Genomic variants with an FDR < 0.005 were classified as statistically significant divergent sites.

In our prior pan-genome study [14], short-read sequencing of 1078 strains and long-read sequencing of 545 strains enabled SNP, InDel, and SV detection. Consequently, in this study, SNP and InDel analyses included the 51 wild, 205 local, 105 CHN-I, and 89 JPN-I strains, while SV analyses comprised 38 wild, 144 local, 67 CHN-I, and 47 JPN-I strains.

For population genetic analysis, we obtained precomputed *F*_ST_ and Tajima’s D statistics from the SilkMeta database [26], as described in our prior pan-genome study [14]. Briefly, selective sweep regions were identified using a 5 kb sliding window (500 bp step) for *F*_ST_, Tajima’s D, and XP-CLR (not present here). Regions overlapping the top 1% *F*_ST_, top 5% XP-CLR, and lowest 5% Tajimas D values (Tajima’s D_descendant_ < Tajima’s D_ancestral_) were defined as candidate selective sweeps. Genes within these regions were prioritized as domestication- or breeding-associated candidates.

Additionally, haplotype inference was performed using DnaSP v6 software [29], and haplotype networks were constructed with PopART v1.7 using the Median-Joining algorithm [30].

### 2.7. Association Study of BmBeta_spc SNPs and Cocoon Traits

We obtained silk production trait data (CW, CSW, and CSR) for 123 silkworm strains from a previous study [14]. SNPs of these strains were retrieved from the SilkMeta database and filtered to exclude sites with minor allele frequencies (MAFs) < 0.05 and missing rates > 5%. The remaining genome-wide SNPs were subjected to principal component analysis (PCA) to assess population structure. SNPs located within the *BmBeta_spc* locus (*n* = 1649) were extracted for association analysis. Association studies between *BmBeta_spc* SNPs and cocoon traits were performed using a linear regression model implemented in PLINK v1.9 [31], with the top four PCA eigenvectors included as covariates to correct for population structure. The significance threshold for *BmBeta_spc* was defined using Bonferroni correction as 0.05/*N* (where *N* is the total number of tested SNPs), yielding a threshold of *p* < 3 × 10^−5^ (−log_10_(*p*) = 4.5).

## 3. Results

### 3.1. Genome-Wide Identification and Chromosomal Distribution

**Figure 1:**
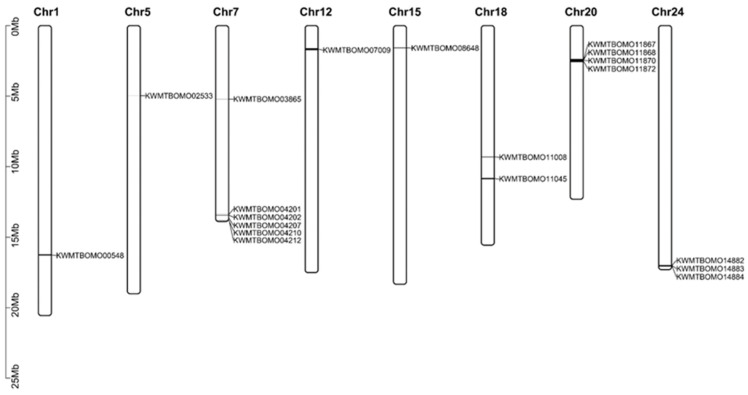
Chromosome distribution of spectrin family genes.

We identified 17 spectrin genes in the silkworm genome by searching SR domains (PF00435) using the HMMER3.0 software package (Table 1). These genes are distributed on eight chromosomes, with KWMTBOMO04201, KWMTBOMO04202, KWMTBOMO04207, KWMTBOMO04210, and KWMTBOMO04212 clustered in Chr7 (Clu1); KWMTBOMO11867, KWMTBOMO11868, KWMTBOMO11870, and KWMTBOMO11872 clustered in Chr20 (Clu2); and KWMTBOMO14882, KWMTBOMO14883, and KWMTBOMO14884 clustered in Chr24 (Clu3) (Figure 1).

In addition to silkworm spectrin genes, we identified 8, 23, and 24 spectrin family genes in the genomes of *D. melanogaster*, *M. musculus*, and *H. sapiens*, respectively (Table S1). While both *B. mori* and *D. melanogaster* are insects, their spectrin gene counts differ significantly. Comparative protein domain analysis revealed fragmented gene predictions in *B. mori*, particularly within the Clu1, Clu2, and Clu3 clusters, indicating incomplete structural annotations. Structural refinement of these genes is therefore essential prior to downstream functional analyses.

### 3.2. Gene Structure Optimization and Characterization of Spectrin Proteins

**Figure 2:**
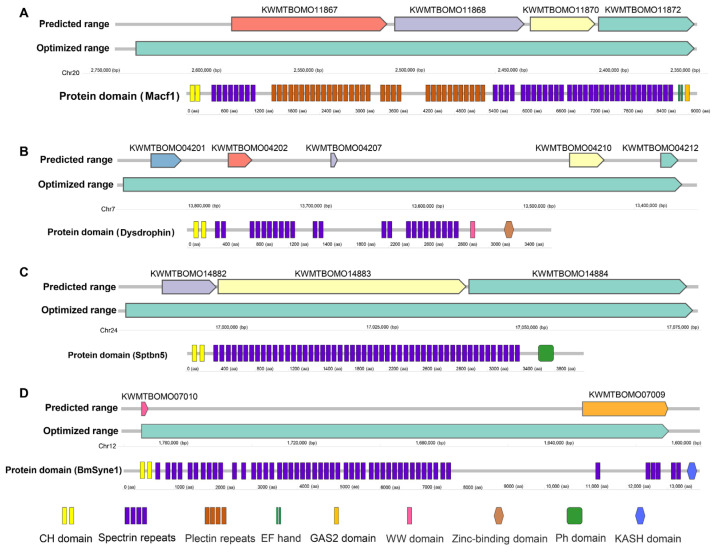
Genomic ranges (predicted ranges and optimized ranges) and protein domains of *BmMacf1* (**A**), *BmDys* (**B**), *BmSptbn5* (**C**), and *BmSyne1* (**D**). CH, Calponin homology domain. GAS2, Growth-Arrest-Specific Protein 2 Domain. WW, Domain with 2 conserved Trp (W) residues. Ph, Pleckstrin homology domain. KASH (for Klarsicht/ANC-1/Syne-1 homology), Nuclear envelope localization domain.

Gene structure prediction accuracy in non-classical model organisms is often suboptimal, particularly for genes encoding large proteins. To address this, we refined spectrin gene structures using transcriptome data, extending transcriptional regions through newly assembled transcripts. Notably, the Clu1, Clu2, and Clu3 clusters were resolved into three distinct transcripts encoding proteins of 3583, 9267, and 4083 amino acids, respectively (Table 1, Figure 2A–C). These were annotated as *BmDys*, *BmMacf1*, and *BmSptbn5*, orthologs of *Dystrophin* (*Dys*), *Macf1*, and *Sptbn5* in other species (Table 2).

Additionally, a transcript linking *KWMTBOMO07009* and *KWMTBOMO07010* encodes a 13,424 aa protein orthologous to *Syne1* (*Msp300* in *Drosophila*, Figure 2D). Four remaining genes were identified as *B. mori* orthologs of *Alpha_spc* (*KWMTBOMO08648*), *Beta_spc* (*KWMTBOMO11045*), *Actn* (*KWMTBOMO11008*), and *Trio* (*KWMTBOMO00548*) (Table 2). Collectively, we identified eight spectrin family genes in the silkworm genome, matching the count in *Drosophila*, indicating conserved gene counts across the two species.

Most spectrin family genes occupy large genomic regions and encode long amino acid sequences. Five genes span over 100 kb, with the largest spanning 501 kb (Table 1). Seven genes encode proteins exceeding 2000 amino acids (aa) (Table 1).

**Figure 3:**
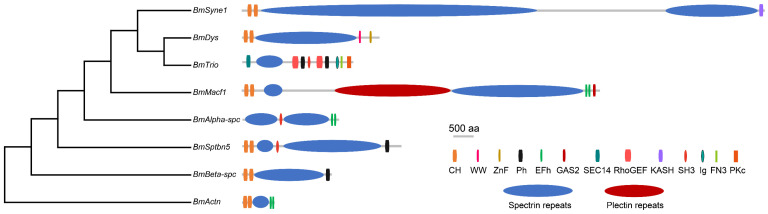
Phylogenetic relationships and protein domains of spectrin family genes in silkworm. CH, Calponin homology domain. WW, Domain with 2 conserved Trp (W) residues. ZnF, Zinc-binding domain. Ph, Pleckstrin homology domain. EFh, EF hand, calcium-binding motif. GAS2, Growth-Arrest-Specific Protein 2 Domain. SEC14, Domain in homologues of a *Saccharomyces cerevisiae* phosphatidylinositol transfer protein. RhoGEF, Guanine nucleotide exchange factor for Rho/Rac/Cdc42-like GTPases. KASH (for Klarsicht/ANC-1/Syne-1 homology), Nuclear envelope localization domain. SH3, Src homology 3 domains. Ig, immunoglobulin. FN3, Src homology 3 domains. PKc, Protein kinase, unclassified specificity.

Protein domain analysis reveals that the majority of the amino acid sequences in spectrin proteins are utilized to form SR domains. BmSyne1 contains 54 SRs, with longer proteins generally having more SRs (Figure 3, Table 1). Additional domains include calponin homology (CH), pleckstrin homology (PH), and EF-hand motifs (EFh) (Figure 3, Table 1). *Macf1* encodes plectin repeat domains, confirming its dual classification within both the spectrin and plectin families.

### 3.3. Phylogenetic Relationships

**Figure 4:**
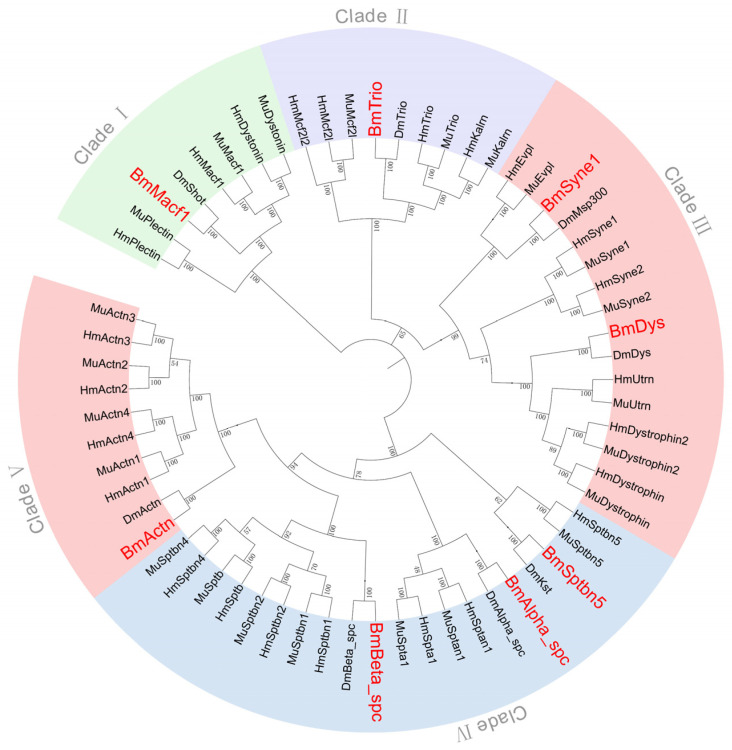
Phylogenetic tree of spectrin family genes across species.

Phylogenetic analysis of spectrin protein sequences from *B. mori*, *D. melanogaster*, *M. musculus*, and *H. sapiens* resolved five distinct clades (Figure 4). Clade I contains *Macf1* (*Shot*) along with *Dystonin* and *Plectin*, which are absent in *B. mori* and *D. melanogaster*. Clade II includes *Trio*, *Kalrn*, *Mcf2l1*, and *Mcf2l2*, with only *Trio* being present in the two insect species. Clade III groups *Syne1* and *Trio* with mammalian homologs (*Syne2*, *Dys2*, *Evpl*, and *Utrn*). Clade IV comprises three spectrins (*Alpha_spec*, *Beta_spec*, and *Sptbn5*) shared across taxa, as well as four mammal-specific genes (*Spta1*, *Sptb*, *Sptbn2*, and *Sptbn4*). Clade V contains five *Actn* homologs (*Actn*, *Actn1*, *Actn2*, *Actn3*, and *Actn4*), with only *Actn* observed in insect genomes.

Mammals exhibit spectrin gene expansions relative to insects. For instance, there are two *Syne* and *Dys* copies, three copies of *Alpha_spc*, and four copies of *Beta_spc* and *Actn* in the two mammals, respectively. This pattern suggests vertebrate-specific functional diversification via gene duplication and dosage-effect evolution.

### 3.4. Spatiotemporal Expression Profiles of Spectrin Genes

Using existing transcriptome data, we analyzed spectrin gene expression across silkworm tissues (hemolymph, epidermis, head, testis, ovary, malpighian tubule, trachea, midgut, fatbody, and silk gland) from day 3 of the fourth larval instar to adulthood. These genes exhibited broad spatiotemporal expression (Figure S1), indicating their essential roles in silkworm growth and development.

*BmActn* is mainly expressed in the larval testis, malpighian tubule, and silk gland, as well as the testis, head, and thorax of the adult. *BmMacf1* has a significantly high expression level in the middle silk gland during the pre-pupal stage. *BmBeta_spc* is expressed in the larval testis, ovary, midgut, as well as the thorax, legs, head, and fatbody of adults, with peak expression in the middle/posterior silk glands during the pre-pupal stage. For *BmTrio*, *BmSptbn5*, and *BmAlpha_spc*, a comparatively higher expression was observed in the majority of the tissues investigated. *BmSptbn5* and *BmAlpha_spc* are generally expressed across different stages of the larval hemolymph, ovary, and malpighian tubule. *BmDys* and *BmSyne1* are mainly expressed in the ovary and malpighian tubule.

### 3.5. BmTrio Plays Roles in Silkworm Domestication

**Figure 5:**
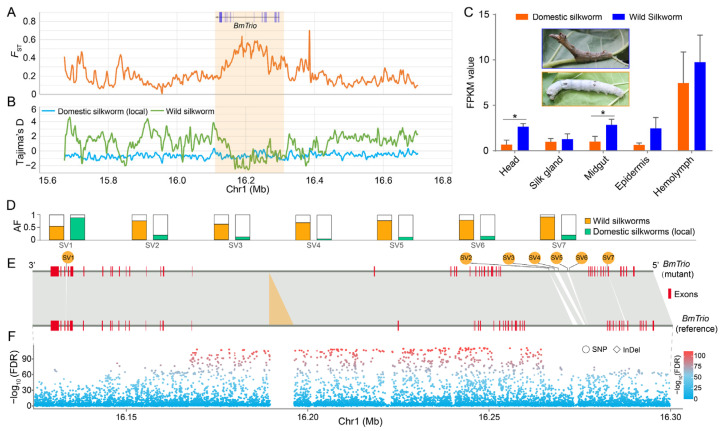
*BmTrio* is associated with silkworm domestication. (**A**) Fixation index (*F*_ST_) values between wild and domesticated (local) silkworm populations. (**B**) Tajima’s D values of wild (green) and local (blue) populations. Orange-shaded areas in (**A**,**B**) indicate the *BmTrio* locus. (**C**) *BmTrio* expression (FPKM) across tissues in wild and domesticated silkworms. Error bars, ± standard deviations (SDs); Student’s *t*-test, * *p* < 0.05. Insets show fifth-instar larvae of wild (blue box) and domesticated (yellow box) silkworms. (**D**) Allele frequencies (AFs) of seven SVs in *BmTrio* exhibiting significant divergence between wild (orange bars) and domesticated (green bars) silkworms. Chi-square test, FDR < 0.005. (**E**) *BmTrio* gene structures with seven SVs (numbered as in (**D**)). Red boxes: exons; orange triangle: a specific insertion in the reference genome. (**F**) The SNPs (circles) and InDels (squares) within the *BmTrio* and its flanking regions. A chi-square test (FDR correction) was applied to evaluate the genetic differentiation of these variants between wild and domesticated silkworms (local population). The color gradient (blue to red) represents the magnitude of log_10_-transformed *p*-values, ranging from 0 (blue, non-significant) to 100 (red, highly significant).

The domesticated silkworm (*B. mori*) derived from its wild counterpart *B. mandarina*, acquiring phenotypic alteration in body color, flight capability, and silk yield during domestication [32,33,34]. Previously, we identified 468 candidate domestication genes, including *BmTrio*, by comparing local and wild silkworm populations [14]. Here, we integrated artificial selection signatures, transcriptome data from diverse tissues on day 3 of the fifth larval instar, and genomic variation analysis of the *BmTrio* locus. We found that the *BmTrio* exhibited strong artificial selection signals during domestication (Figure 5A,B). The expression level of *BmTrio* in various tissues of wild silkworms is higher than that in domesticated silkworms (Figure 5C). Specifically, significant differences (Student’s *t*-test, *p* < 0.05) were observed in the head and midgut (Figure 5C). In the genomic and flanking regions of *BmTrio* (within a 5 kb range both upstream of the transcriptional start site and downstream of the transcriptional end site), we discovered 11,573 SNPs, 2199 InDels, and 381 SVs (Table S2). Of these, 7350 SNPs, 1161 InDels, and 7 SVs exhibited significant divergence (chi-square test, FDR < 0.005) between wild and local silkworm populations (Figure 5D–F, Table S2). These divergent genetic variations in *BmTrio* underscore its pronounced divergence during domestication. Its downregulation in domesticated silkworms likely stems from selection on linked regulatory variants, driving phenotypic adaptation.

### 3.6. BmBeta_spc Is Associated with Silk Yield

The domesticated silkworm has undergone intensive selective breeding over the past century, yielding Chinese- (CHN-I) and Japanese-improved (JPN-I) strains with enhanced silk production. Our prior pan-genome study identified 126 and 116 genes associated with the breeding of CHN-I and JPN-I, respectively [14]. *BmBeta_spc* is one of the genes associated with CHN-I improvement.

**Figure 6:**
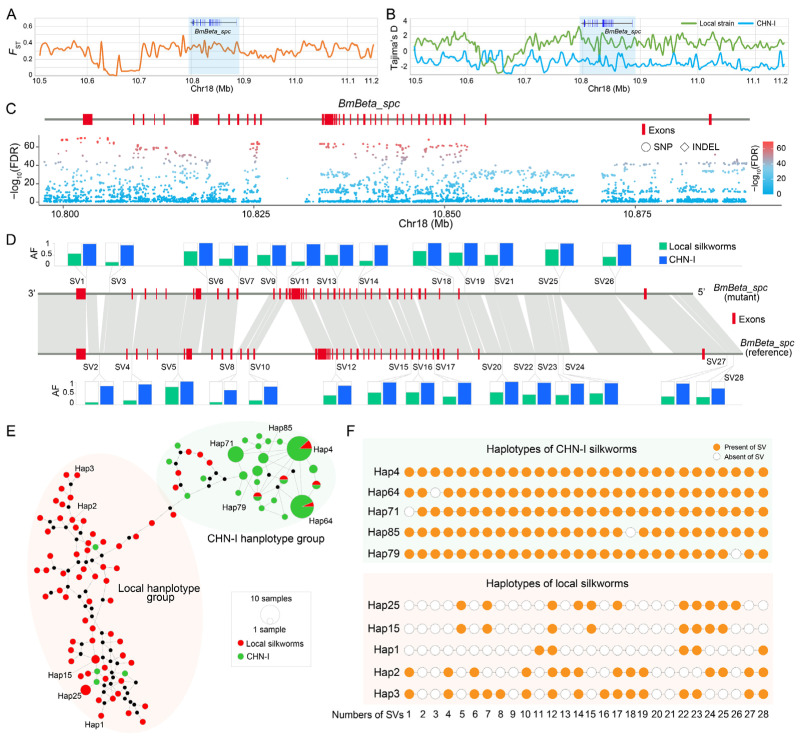
*BmBeta_spc* genomic variations diverge between local and CHN-I silkworm subpopulations. (**A**) Fixation indices (*F*_ST_) between local and CHN-I subpopulations. (**B**) Tajima’s D values for local (green) and CHN-I (blue) silkworms. Blue-shaded regions in (**A**,**B**) mark the *BmBeta_spc* locus. (**C**) SNPs (circles) and InDels (squares) within the *BmBeta_spc* genomic region and its flanking regions are shown. The chi-square test (FDR-corrected) was applied to evaluate the genetic differentiation of these variants between local and CHN-I silkworms. The color gradient (blue to red) represents the magnitude of log_10_-transformed *p*-values, ranging from 0 (blue, non-significant) to 100 (red, highly significant). (**D**) Allele frequencies (AFs) of 28 SVs in *BmBeta_spc* exhibiting significant divergence between local (green) and CHN-I (blue) silkworms. Chi-square test, FDR < 0.005. (**E**) Haplotype network of 28 SVs in local and CHN-I individuals. (**F**) Ten representative haplotypes in local and CHN-I subpopulations. Orange circles: SV presence; white dashed circles: SV absence. SV numbering matches panel D. These representative haplotypes were labeled in the haplotype network of panel (**E**).

Population genomic analyses revealed strong selection signatures at the *BmBeta_spc* locus, evidenced by elevated *F*_ST_ (fixation index) and divergent Tajima’s D statistics between local and CHN-I silkworm subpopulations (Figure 6A,B). Utilizing our previously published silkworm pan-genome dataset, we identified 5016 genetic variations in the *BmBeta_spc* locus and its flanking region (Table S3). These included 3913 SNPs, 772 InDels, and 331 SVs (Table S3). Of these variations, 1945 SNPs, 162 InDels, and 28 SVs show significant divergence (chi-square test, FDR < 0.005) between local and CHN-I subpopulations (Figure 6C,D; Table S3), predominantly in intronic and regulatory regions (Figure 6C,D; Table S3). Haplotype analysis of 28 SVs across 141 silkworms (74 local, 67 CHN-I) resolved 93 haplotypes (Figure 6E, Table S4), which mainly clustered into two groups, the local and CHN-I haplotype groups (Figure 6E, Table S4). The local haplotypes display higher diversity and carry fewer SVs compared to the CHN-I haplotypes (Figure 6E,F). For instance, within the CHN-I haplotype group, hap4 contains all 28 SVs, and hap64, hap71, hap85, and hap79 encompass 27 SVs. In contrast, the haplotypes of the local haplotype group lack about half or more of the SVs (Figure 6F, Table S4). These findings suggest positive selection of *BmBeta_spc* during silkworm breeding.

**Figure 7:**
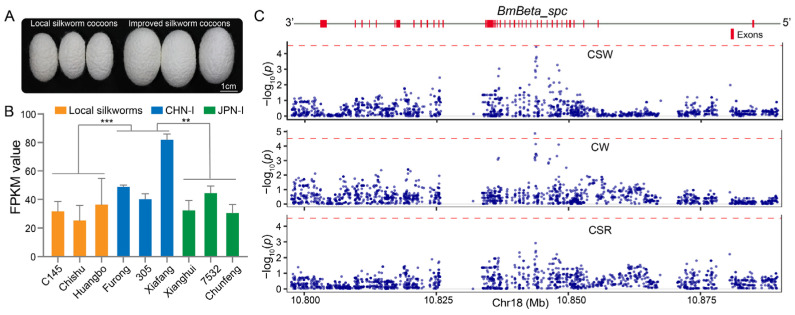
*BmBeta_spc* is associated with silkworm breeding traits. (**A**) Cocoon size comparison between local (smaller) and improved silkworm strains. (**B**) *BmBeta_spc* expression (FPKM) in silk glands of local (C145, Chishu, and Huangbo), CHN-I (Furong, 305, and Xiafang), and JPN-I (Xianghui, 7532, and Chunfeng) silkworm strains. Error bars, ± standard deviations (SDs); Student’s *t*-test, ** *p* < 0.01, *** *p* < 0.001. (**C**) Associations of *BmBeta_spc* SNPs with CSW, CW, and CSR. Red dashed lines: the significance threshold for *BmBeta_spc* that was defined using Bonferroni correction as 0.05/*N* (where *N* is the total number of tested SNPs).

Additionally, we performed a comparative transcriptome analysis of the silk gland, an organ responsible for silk protein synthesis and storage, in local (C145, Chishu, and Huangbo strains), CHN-I (Furong, 305, and Xiafang strains), and JPN-I (Xianghui, 7532, and Chunfeng strains) silkworms (Figure 7A). This analysis demonstrated significantly elevated *BmBeta_spc* expression in CHN-I silkworms relative to JPN-I and local strains (Figure 7B). Furthermore, we employed SNPs (*n* = 1649) within the genomic region of *BmBeta_spc* to conduct an association analysis with silkworm cocoon yield traits, including cocoon weight (CW), cocoon shell weight (CSW), and cocoon shell ratio (CSR) (Figure S2 and Figure 3). Significant associations were identified between *BmBeta_spc* SNPs and cocoon traits (CSW and CW) (Figure 7C). The SNP bomsnp28075049 exhibited a moderate effect on CSW (*β* = 0.03, *p* = 3.66 × 10^−5^), while SNP bomsnp28075021 showed a stronger cumulative effect on CW (mean *β* = 0.115, *p* = 1.35 × 10^−5^) (Table S5, Figure 7C). These results suggest that *BmBeta_spc* may serve as a promising target for enhancing silk yield during silkworm breeding.

### 3.7. BmBeta_spc Potentially Contributes to Heterosis of Silk Yield

In animal and plant breeding, cross-breeding is a prevalent method for developing new varieties by leveraging heterosis. In silkworms, CHN-I and JPN-I strains are recognized as the two subpopulations exhibiting a high heterosis efficiency [35]. Our transcriptome analysis demonstrated significantly higher *BmBeta_spc* expression in the silk glands of CHN-I silkworms compared to those of JPN-I strains (Figure 7B).

**Figure 8:**
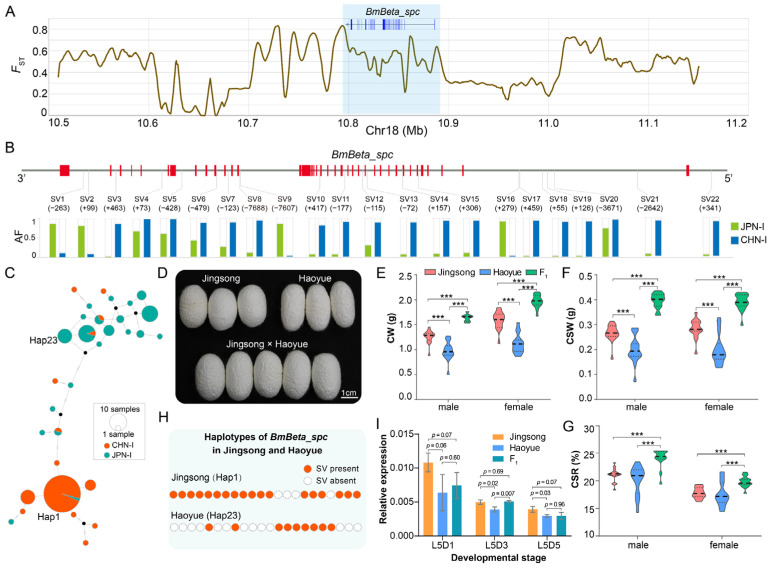
*BmBeta_spc* contributes to silk yield heterosis. (**A**) Fixation indices (*F*_ST_) between CHN-I and JPN-I subpopulations. Blue-shaded region marks the *BmBeta_spc* locus. (**B**) *BmBeta_spc* gene structure, with 22 SVs showing significant allele frequency (AF) divergence (CHN-I: blue; JPN-I: green). Chi-square test, FDR < 0.005. Numbers in parentheses denote structural variation (SV) sizes in base pairs (bp), with “+” indicating insertions and “−” representing deletions. (**C**) Haplotype network of 22 SVs. (**D**) Cocoon phenotypes of Jingsong and Haoyue and their F_1_ hybrids. (**E**–**G**) Silk yield traits (CW, CSR, and CSR) in parents and F_1_ hybrids. Student’s *t*-test, *** *p* < 0.001. (**H**) SV haplotypes in Jingsong and Haoyue. Orange circles: SV presence; white dashed circles: SV absence. SV order matches panel (**B**). (**I**) *BmBeta_spc* expression in parental and F_1_ hybrids at days 1, 3, and 5 of the fifth larval instar, denoted as L5D1, L5D3, and L5D5. Error bars, ± standard deviations (SDs); Student’s *t*-test.

To investigate the association of this gene with silkworm heterosis, we analyzed population differentiation between CHN-I and JPN-I strains. The result showed that the *F*_ST_ value in the *BmBeta_spc* genomic region exceeded 0.8 (Figure 8A). Comparative analysis of allele frequencies in the gene and flanking regions identified 1857 significantly differentiated variations (chi-square test, FDR < 0.005), including 1590 SNPs, 262 InDels, and 22 SVs (Figure 8B, Table S6). Haplotype analysis of these SVs in 67 CHN-I and 51 JPN-I silkworms showed that CHN-I and JPN-I strains were clustered into two main clades (Figure 8C). JPN-I strains comprised 22 haplotypes, while CHN-I strains exhibited 9 haplotypes, with 45 CHN-I strains sharing a single dominant haplotype (hap1) (Figure 8C, Table S7). These results indicate substantial genetic differentiation in *BmBeta_spc* between the two subpopulations, potentially explaining its expression differences in silk glands.

The hybrid cross “Jingsong × Haoyue”, a widely used commercial variety in Chinese sericulture, showed significant heterosis in silk yield traits. The F_1_ offspring exhibited significantly higher CW, CSW, and CSR values than either parent (Figure 8D–G), showing overdominant characteristics. The SV haplotype of *BmBeta_spc* in Jingsong (a CHN-I strain) differed from Haoyue (a JPN-I strain), consistent with the CHN-I/JPN-I divergence (Figure 8C,H). A qPCR analysis of silk glands from both parents and F_1_ offspring during the fifth larval stage (days 1, 3, and 5) confirmed lower *BmBeta_spc* expression in Haoyue than in Jingsong (Figure 8I), aligning with expression trends observed in other CHN-I and JPN-I silkworms (Figure 7B). Notably, F_1_ offspring exhibited expression levels comparable to those of the Haoyue parent (both slightly below Jingsong) on days 1 and 5 of the fifth larval instar, while showing significantly higher expression (approaching Jingsong levels) on day 3 (Figure 8I). This expression pattern aligns with the dominant inheritance model underlying heterosis [28]. These findings suggest that *BmBeta_spc* may contribute to silk yield heterosis in hybrid silkworms.

## 4. Discussion

The spectrin family genes, encoding large multidomain proteins critical for cytoskeletal organization, have been extensively characterized in model organisms, demonstrating pleiotropic roles across diverse tissues, including nervous systems, musculature, and embryonic development [36,37,38]. In this study, we systematically investigated the genomic architecture and functional implications of spectrin genes in silkworm domestication and breeding. Initial identification using SRs as signature domains revealed 17 predicted genes in the silkworm genome. Subsequent domain analysis exposed fragmented annotations, particularly for ultra-large genes (>8000 amino acids) like *Macf1*—a known challenge for conventional gene prediction algorithms [39]. Through transcriptome-guided structural refinement, we resolved eight functional spectrin genes (*BmDys*, *BmMacf1*, *BmSptbn5*, et al.), correcting assembly errors from fragmented predictions. This computational pipeline addresses gene annotation inaccuracies through transcriptome-guided structural refinement, providing an alternative strategy for optimizing gene predictions in other non-classical model organisms.

Multi-species genomic analysis identified orthologs of these silkworm spectrins in *H. sapiens*, *M. musculus*, and *D. melanogaster*. Notably, mammalian genomes exhibited gene expansions, containing 23 (*M. musculus*) and 24 (*H. sapiens*) spectrin paralogs compared to 8 in *D. melanogaster* and *B. mori*. This pattern suggests deep evolutionary conservation of core spectrin functions across metazoans, alongside functional divergence in vertebrates. Spectrin genes were first isolated from erythrocyte ghosts and then were found in non-erythroid tissues, such as the brain and muscle [4,5]. Spatiotemporal expression profiles in silkworm reveal a multi-tissue expression pattern of spectrin family genes, extending our understanding of their potential roles in insect development.

Among the eight spectrin family genes in the silkworm genome, *BmTrio* exhibited pronounced selection signatures during silkworm domestication, with elevated expression in the heads and midguts of wild silkworms (*B. mandarina*). The larval head of the silkworm contains organs including the cerebral ganglia, olfactory sensory receptors, salivary glands, and the spinneret. The cerebral ganglia regulate behaviors like mimicry (cryptic behaviors mimicking tree branches) and modulate hormone secretion [40,41], thereby influencing growth and development. The olfactory sensory receptors govern feeding preferences [42], while salivary glands facilitate food digestion, and the spinneret plays a pivotal role in silk fiber formation [43]. During domestication, silkworms exhibit reduced locomotor activity, loss of mimicry behavior, and altered olfactory perception [34,40,44]. Given the conserved role of *BmTrio* in neural development across species (e.g., *Drosophila* and mammals) [45,46], we hypothesize that it may contribute to neural development in silkworms, potentially influencing domestication-related traits. Nevertheless, the functional roles of this gene in silkworm domestication require systematic experimental validation using targeted functional genomic approaches.

Through integrative analyses of artificial selection signatures, phenotype–genotype associations, and expression profiles, we demonstrated that *BmBeta_spc* is a potential target for silk yield enhancement, underscoring its agricultural relevance. Notably, we identified a number of SNPs, InDels, and SVs that associated with silkworm breeding and silk yield. These variants could be markers for marker-assisted breeding aimed at improving silk production.

Summarily, this study expands the functional characterization of spectrin family genes to a non-classical model organism, the silkworm *Bombyx mori*. Our findings underscore the expression of these genes across diverse tissues, including the head, midgut, and silk gland. We propose mechanistic roles for *BmTrio* in domestication-associated behavioral adaptations and *BmBeta_spc* in silk yield heterosis. Future work should prioritize functional validation using CRISPR/Cas9-mediated knockout to elucidate their contributions to silk gland development, fibroin biosynthesis, and neurobehavioral regulations.

## Figures and Tables

**Table 1:** Genomic ranges and protein domains of spectrin family genes.

Gene ID	Chr.	Strand (+/−)	Predicted Genomic Region	Optimized Genomic Region	AAS Length (aa)	Protein Domain
KWMTBOMO14882	24	+	16986141–16994985	16927869–17074342	4083	Spectrin repeats (29), PH, CH
KWMTBOMO14883	24	+	16995271–17036415			
KWMTBOMO14884	24	+	16995271–17036415			
KWMTBOMO11867	20	−	2360655–2407092	2359470–2714880	9267	GAS2, plectin repeats (36), spectrin repeats (34), CH
KWMTBOMO11868	20	−	2408554–2440562			
KWMTBOMO11870	20	−	2442893–2507042			
KWMTBOMO11872	20	−	2510156–2587372			
KWMTBOMO04201	7	−	13360093–13375354	13357711–13859261	3583	Spectrin repeats (15), WW, ZnF, CH
KWMTBOMO04202	7	−	13427021–13458274			
KWMTBOMO04207	7	−	13666034–13671749			
KWMTBOMO04210	7	−	13743571–13764629			
KWMTBOMO04212	7	−	13807018–13833429			
KWMTBOMO11045	18	−	10803357–10884353	10802397–10884586	2575	Spectrin repeats (17), PH, CH
KWMTBOMO08648	15	−	1561077–1596812	1560551–1596880	2421	Spectrin repeats (19), EFh
KWMTBOMO00548	1	−	16223310–16295639	16129442–16295639	2851	Spectrin repeats (7), SEC14, RhoGEF, PH
KWMTBOMO07009	12	−	1611446–1737463	1610900–1774453	13424	Spectrin repeats (54), KASH, CH
KWMTBOMO11008	18	−	9286104–9331592	9285843–9331723	899	Spectrin repeats (4), EFh, CH

**Table 2:** Orthologs of spectrin genes in various species.

Gene ID	*B. mori*	Orthologs		
		*D. melanogaster*	*H. sapiens*	*M. musculus*
KWMTBOMO14882/14883/14884	*BmSptbn5*	*Karst*	*Sptbn5*	*Sptbn5*
KWMTBOMO11867/118688/11870	*BmMacf1*	*Short stop*	*Macf1*	*Macf1*
KWMTBOMO04201/04202/04207/04210/04212	*BmDys*	*Dys/dystrophin*	*Dystrophin*	*Dystrophin*
KWMTBOMO11045	*BmBeta_spc*	*Beta spectrin*	*Sptbn1*	*Sptbn1*
KWMTBOMO08648	*BmAlpha_spc*	*Alpha spectrin*	*Sptan1*	*Sptan1*
KWMTBOMO00548	*BmTrio*	*Trio*	*Trio*	*Trio*
KWMTBOMO07009	*BmSyne1*	*Msp300*	*Syne1*	*Syne1*
KWMTBOMO11008	*BmActn*	*Actn*	*Actn1*	*Actn1*

## Data Availability

The mRNA and protein sequences of spectrin family genes were deposited in the Genebase database (https://ngdc.cncb.ac.cn/genbase, accessed on 3 May 2025) maintained by the China National Center for Bioinformation (CNCB) under accession numbers C_AA107949.1 to C_AA107956.1. The raw RNA-seq data used for gene structure refinement are accessible via the Sequence Read Archive (SRA) database (https://www.ncbi.nlm.nih.gov/sra, accessed on 2 January 2020) under sample IDs SRR10035668 and SRR10035660. Spatiotemporal expression profiles were generated from transcriptome data in BioProject PRJNA559726. Silk gland transcriptomes for comparative analysis are available in the Genome Sequence Archive (GSA, https://ngdc.cncb.ac.cn/gsa/, accessed on 20 February 2025) under accession number CRA007878. Genomic variations are available through the CNGB Nucleotide Sequence Archive (CNSA, https://db.cngb.org, accessed on 19 February 2025) under BioProject CNP0001815. All raw data are also accessible via the download module of the SilkMeta database (http://silkmeta.org.cn, accessed on 19 February 2025) using project IDs, sample IDs, or strain names.

## Supplementary Materials

The following supporting information can be downloaded at https://www.mdpi.com/article/10.3390/insects16060556/s1: Figure S1: Spatial and temporal expression of spectrin family genes in silkworm; Figure S2: Principal component analysis (PCA) based on genome-wide SNP data from 123 silkworm strains; Figure S3: Frequency distribution analysis of cocoon yield traits; Table S1: Spectrin family genes across species (*B. mori*, *D. melanogaster*, *M. musculus*, and *H. sapiens*); Table S2: Differentiation of genetic variations (SNPs, InDels, and SVs) in the *BmTrio* locus between wild and domesticated (local) silkworms; Table S3: Differentiation of genetic variations (SNPs, InDels, and SVs) in the *BmBeta_spc* locus between local and CHN-I silkworms; Table S4: Haplotypes of 28 SVs in the *BmTrio* locus between local and CHN-I silkworms; Table S5: Summary of the association signals between *BmBeta_spc* SNPs and cocoon traits (CW, CSW, and CSR); Table S6: Differentiation of genetic variations (SNPs, InDels, and SVs) in the *BmBeta_spc* locus between JPN-I and CHN-I silkworms; Table S7: Haplotypes of 22 SVs in the *BmBeta_spc* locus between JPN-I and CHN-I silkworms.
