# Supplementary material for: Characterization of Spectrin Family Genes and Their Evolutionary Roles in Domestication and Breeding of the Silkworm Bombyx mori
Source: Insects. 2025 May 24;16(6):556. doi: 10.3390/insects16060556 (PMC12193018; doi:10.3390/insects16060556)
Supplement: Supplementary file 1 [file insects-16-00556-s001.zip › Supplementary figures.pdf]

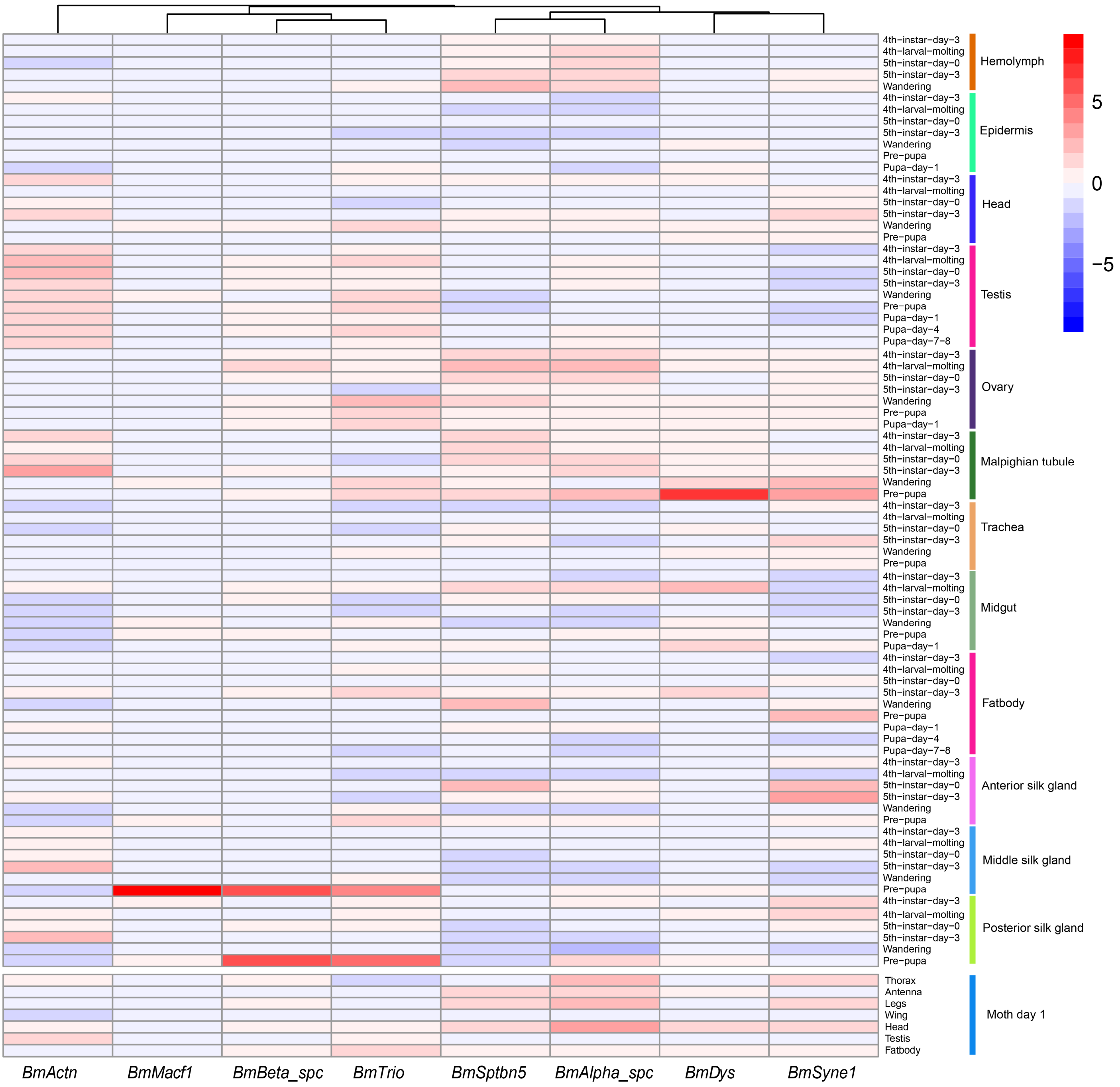

Figure S1. Spatio and temporal expression of spectrin family genes in silkworm

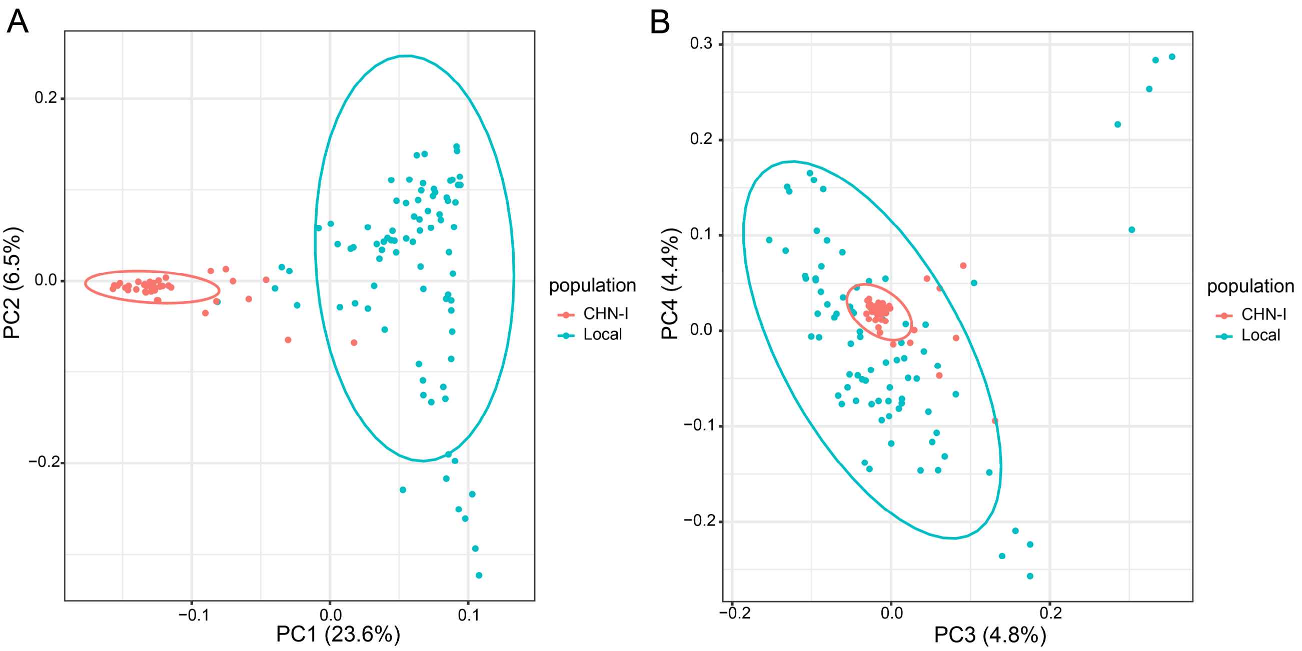

Figure S2. Principal component analysis (PCA) based on genome-wide SNP data from 123 silkworm strains. (A) Scatter plot showing the distribution of samples along PC1 and PC2 axes. (B) Pairwise comparison of PC3 and PC4 components. Chinese-improved silkworm strains are highlighted in red, while indigenous local silkworm populations are depicted in green.

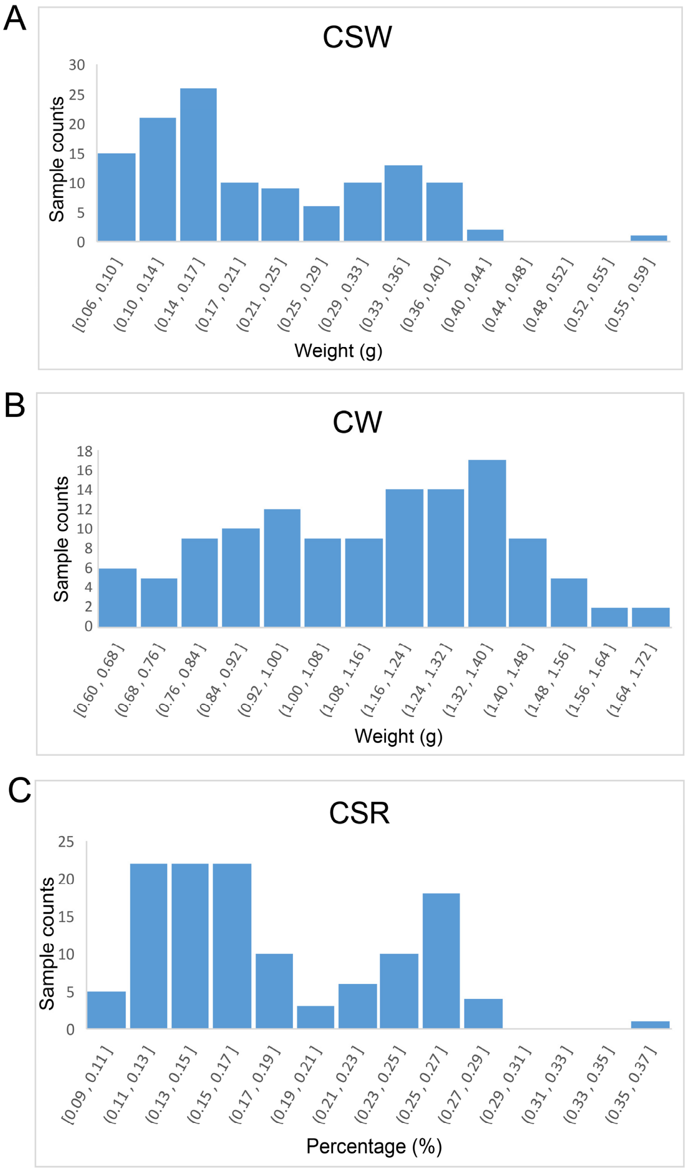

Figure S3. Frequency distribution analysis of cocoon yield traits. (A) Histogram of cocoon shell weight (CSW). (B) Distribution profile of cocoon weight (CW). (C) Statistical spread of cocoon shell ratio (CSR; CSR = CSW/CW × 100%).
